# Bis(2-ethyl-1*H*-imidazol-3-ium) tetra­chloridomercurate(II)

**DOI:** 10.1107/S1600536811055371

**Published:** 2012-01-14

**Authors:** Run-Qiang Zhu

**Affiliations:** a Ordered Matter Science Research Center, College of Chemistry and Chemical Engineering, Southeast University, Nanjing 211189, People’s Republic of China

## Abstract

The crystal structure of the title compound, (C_5_H_9_N_2_)_2_[HgCl_4_], consists of discrete tetra­chloridomercurate dications and discrete 2-methyl­imidazolium cations. In the complex anion, the mercury cations are coordinated by four chloride anions with distances between 2.4568 (14) and 2.4936 (15) Å in a tetra­hedral geometry. In the crystal, the cations and anions are connected by inter­molecular N—H⋯Cl inter­actions. One C atom of the cations is disordered and was refined using a split model (occupancy ratio 0.75:0.25).

## Related literature

For a related structure and background to this study, see: Zhu (2011 ▶).
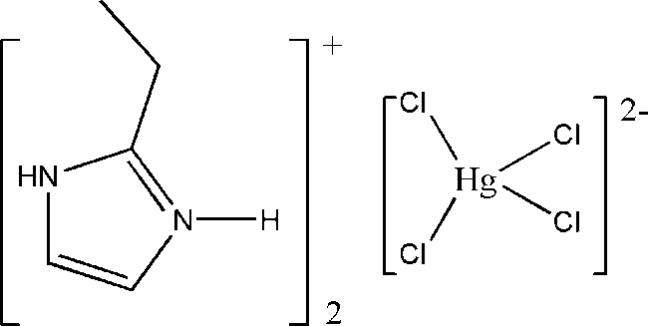


## Experimental

### 

#### Crystal data

(C_5_H_9_N_2_)_2_[HgCl_4_]
*M* *_r_* = 536.67
Triclinic, 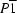

*a* = 7.5784 (15) Å
*b* = 8.0972 (16) Å
*c* = 14.661 (3) Å
α = 92.42 (3)°
β = 97.88 (3)°
γ = 98.17 (3)°
*V* = 880.4 (3) Å^3^
*Z* = 2
Mo *K*α radiation
μ = 9.34 mm^−1^
*T* = 293 K
0.33 × 0.28 × 0.20 mm

#### Data collection

Rigaku SCXmini diffractometer
Absorption correction: multi-scan (*CrystalClear*; Rigaku, 2005 ▶) *T*_min_ = 0.216, *T*_max_ = 0.459
9149 measured reflections
4032 independent reflections
3437 reflections with *I* > 2σ(*I*)
*R*_int_ = 0.044

#### Refinement

*R*[*F* ^2^ > 2σ(*F* ^2^)] = 0.036
*wR*(*F* ^2^) = 0.064
*S* = 1.05
4032 reflections
178 parameters
H-atom parameters constrained
Δρ_max_ = 0.80 e Å^−3^
Δρ_min_ = −0.72 e Å^−3^

### 

Data collection: *CrystalClear* (Rigaku, 2005 ▶); cell refinement: *CrystalClear*; data reduction: *CrystalClear*; program(s) used to solve structure: *SHELXS97* (Sheldrick, 2008 ▶); program(s) used to refine structure: *SHELXL97* (Sheldrick, 2008 ▶); molecular graphics: *DIAMOND* (Brandenburg & Putz, 2005 ▶); software used to prepare material for publication: *SHELXL97*.

## Supplementary Material

Crystal structure: contains datablock(s) I, global. DOI: 10.1107/S1600536811055371/nc2260sup1.cif

Structure factors: contains datablock(s) I. DOI: 10.1107/S1600536811055371/nc2260Isup2.hkl

Additional supplementary materials:  crystallographic information; 3D view; checkCIF report

## Figures and Tables

**Table 1:** Hydrogen-bond geometry (Å, °)

*D*—H⋯*A*	*D*—H	H⋯*A*	*D*⋯*A*	*D*—H⋯*A*
N1—H1*N*⋯Cl3^i^	0.86	2.42	3.227 (4)	157
N2—H2*N*⋯Cl4^ii^	0.86	2.37	3.188 (4)	158
N3—H3*N*⋯Cl2	0.86	2.37	3.220 (4)	171
N4—H4*N*⋯Cl2^iii^	0.86	2.47	3.285 (4)	159

Symmetry codes: (i) 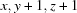; (ii) 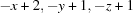; (iii) 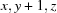.

**Fig. 1.**
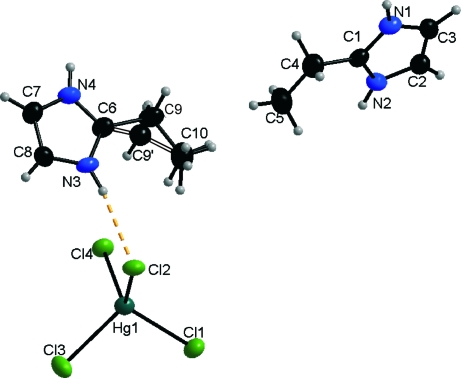
The molecular structure of the title compound, with labeling and displacement ellipsoids drawn at the 30% probability level. Intermolecular hydrogen bonding is shown as dashed lines and disordering as full and open bonds..

**Fig. 2.**
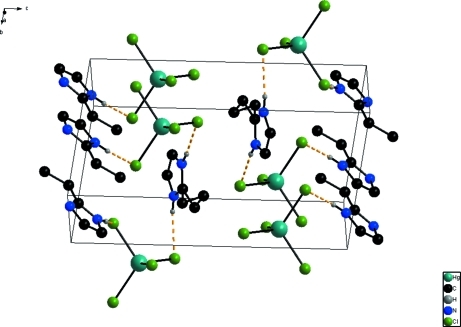
Crystal structure of the title compound with intermolecular hydrogen bonding shown as dashed lines. A-atoms not involved in hydrogen bonding are omitted for clarity.

## supplementary crystallographic information

### Experimental

A mixture of HgCl_2_ (4.26 g, 25 mmol), hydrochloric acid (50 mmol, 36%, 8 ml), and 2 - ethyl imidazole (4.8 g, 50 mmol) in 30 ml water was stirred for 10 minutes at room temperature. On slow evaporation of solvent colourless crystals of the title compound grew within two weeks.

### Refinement

Hydrogen atom positions were calculated and allowed to ride on their respective C atoms and N atoms with C–H distances of 0.93–0.97Å and N–H = 0.86 Å, and with *U*_iso_(H)=1.2*U*_eq_(C or N) or or 1.5 *U*_iso_(C) for methy H atoms. One C atom is disordered and was refined using a split model and sof of 0.75:0.25. The atom of lower occupany was refined isotropic.

### Crystal data

**Table:**

(C_5_H_9_N_2_)_2_[HgCl_4_]	*Z* = 2
*M**_r_* = 536.67	*F*(000) = 508
Triclinic, *P*1	*D*_x_ = 2.025 Mg m^−^^3^
Hall symbol: -P 1	Mo *K*α radiation, λ = 0.71073 Å
*a* = 7.5784 (15) Å	Cell parameters from 4032 reflections
*b* = 8.0972 (16) Å	θ = 2.3–27.5°
*c* = 14.661 (3) Å	µ = 9.34 mm^−^^1^
α = 92.42 (3)°	*T* = 293 K
β = 97.88 (3)°	Block, colourless
γ = 98.17 (3)°	0.33 × 0.28 × 0.20 mm
*V* = 880.4 (3) Å^3^	

### Data collection

**Table:**

Rigaku SCXmini diffractometer	3437 reflections with *I* > 2σ(*I*)
Radiation source: fine-focus sealed tube	*R*_int_ = 0.044
graphite	θ_max_ = 27.5°, θ_min_ = 3.3°
ω scans	*h* = −9→9
Absorption correction: multi-scan (*CrystalClear*; Rigaku, 2005)	*k* = −10→10
*T*_min_ = 0.216, *T*_max_ = 0.459	*l* = −19→19
9149 measured reflections	2 standard reflections every 150 reflections
4032 independent reflections	intensity decay: none

### Refinement

**Table:**

Refinement on *F*^2^	Secondary atom site location: difference Fourier map
Least-squares matrix: full	Hydrogen site location: inferred from neighbouring sites
*R*[*F*^2^ > 2σ(*F*^2^)] = 0.036	H-atom parameters constrained
*wR*(*F*^2^) = 0.064	*w* = 1/[σ^2^(*F*_o_^2^) + (0.0134*P*)^2^] where *P* = (*F*_o_^2^ + 2*F*_c_^2^)/3
*S* = 1.05	(Δ/σ)_max_ = 0.001
4032 reflections	Δρ_max_ = 0.80 e Å^−^^3^
178 parameters	Δρ_min_ = −0.72 e Å^−^^3^
0 restraints	Extinction correction: *SHELXL97* (Sheldrick, 2008), Fc^*^=kFc[1+0.001xFc^2^λ^3^/sin(2θ)]^-1/4^
Primary atom site location: structure-invariant direct methods	Extinction coefficient: 0.0113 (4)

### Special details

**Table:**

Geometry. All e.s.d.'s (except the e.s.d. in the dihedral angle between two l.s. planes) are estimated using the full covariance matrix. The cell e.s.d.'s are taken into account individually in the estimation of e.s.d.'s in distances, angles and torsion angles; correlations between e.s.d.'s in cell parameters are only used when they are defined by crystal symmetry. An approximate (isotropic) treatment of cell e.s.d.'s is used for estimating e.s.d.'s involving l.s. planes.
Refinement. Refinement of *F*^2^ against ALL reflections. The weighted *R*-factor *wR* and goodness of fit *S* are based on *F*^2^, conventional *R*-factors *R* are based on *F*, with *F* set to zero for negative *F*^2^. The threshold expression of *F*^2^ > σ(*F*^2^) is used only for calculating *R*-factors(gt) *etc*. and is not relevant to the choice of reflections for refinement. *R*-factors based on *F*^2^ are statistically about twice as large as those based on *F*, and *R*- factors based on ALL data will be even larger.

### Fractional atomic coordinates and isotropic or equivalent isotropic displacement parameters (Å^2^)

**Table:**

	*x*	*y*	*z*	*U*_iso_*/*U*_eq_	Occ. (<1)
Hg1	0.79183 (2)	0.20862 (2)	0.266500 (12)	0.05040 (10)	
C1	0.8846 (6)	0.7508 (6)	1.0072 (3)	0.0491 (11)	
C2	1.1813 (6)	0.8008 (6)	1.0293 (3)	0.0573 (13)	
H2	1.3004	0.8002	1.0206	0.069*	
C3	1.1239 (7)	0.8776 (7)	1.0993 (3)	0.0585 (13)	
H3	1.1950	0.9403	1.1492	0.070*	
C4	0.6932 (6)	0.6871 (7)	0.9695 (4)	0.0713 (16)	
H4A	0.6255	0.7802	0.9668	0.086*	
H4B	0.6434	0.6101	1.0116	0.086*	
C5	0.6688 (8)	0.6002 (8)	0.8757 (4)	0.0884 (19)	
H5A	0.7108	0.6775	0.8327	0.133*	
H5B	0.5434	0.5589	0.8567	0.133*	
H5C	0.7365	0.5085	0.8775	0.133*	
C6	0.5651 (7)	0.7388 (7)	0.3880 (4)	0.0593 (13)	
C7	0.2790 (7)	0.7388 (7)	0.3359 (3)	0.0616 (14)	
H7	0.1715	0.7778	0.3157	0.074*	
C8	0.3024 (7)	0.5809 (7)	0.3409 (3)	0.0594 (13)	
H8	0.2153	0.4872	0.3255	0.071*	
C9	0.7550 (11)	0.8057 (10)	0.4345 (7)	0.079 (3)	0.75
H9A	0.8126	0.8820	0.3945	0.095*	0.75
H9B	0.7478	0.8690	0.4912	0.095*	0.75
C9'	0.771 (4)	0.770 (4)	0.3870 (18)	0.066 (8)*	0.25
H9C	0.7969	0.7341	0.3268	0.080*	0.25
H9D	0.8152	0.8883	0.3977	0.080*	0.25
C10	0.8631 (8)	0.6817 (9)	0.4553 (5)	0.099 (2)	
H10A	0.9809	0.7330	0.4838	0.149*	0.75
H10B	0.8729	0.6193	0.3995	0.149*	0.75
H10C	0.8097	0.6078	0.4968	0.149*	0.75
H10D	0.9795	0.7445	0.4775	0.149*	0.25
H10E	0.8776	0.5746	0.4291	0.149*	0.25
H10F	0.7946	0.6664	0.5055	0.149*	0.25
N1	0.9390 (5)	0.8458 (5)	1.0834 (3)	0.0551 (10)	
H1N	0.8690	0.8828	1.1183	0.066*	
N2	1.0314 (5)	0.7232 (5)	0.9728 (3)	0.0530 (10)	
H2N	1.0323	0.6648	0.9225	0.064*	
N3	0.4807 (6)	0.5828 (5)	0.3731 (3)	0.0577 (11)	
H3N	0.5303	0.4948	0.3828	0.069*	
N4	0.4401 (6)	0.8336 (5)	0.3658 (3)	0.0570 (11)	
H4N	0.4583	0.9411	0.3696	0.068*	
Cl1	1.08854 (17)	0.11817 (17)	0.30103 (10)	0.0661 (4)	
Cl2	0.62204 (16)	0.22853 (13)	0.39872 (8)	0.0516 (3)	
Cl3	0.60382 (15)	−0.01229 (18)	0.15686 (8)	0.0652 (4)	
Cl4	0.84770 (16)	0.47931 (15)	0.19336 (8)	0.0567 (3)	

### Atomic displacement parameters (Å^2^)

**Table:**

	*U*^11^	*U*^22^	*U*^33^	*U*^12^	*U*^13^	*U*^23^
Hg1	0.05255 (14)	0.04618 (14)	0.05352 (14)	0.00583 (9)	0.01238 (9)	0.00504 (9)
C1	0.048 (3)	0.043 (3)	0.060 (3)	0.010 (2)	0.014 (2)	0.014 (2)
C2	0.048 (3)	0.058 (3)	0.066 (3)	0.006 (2)	0.010 (3)	0.007 (3)
C3	0.065 (3)	0.051 (3)	0.055 (3)	0.002 (3)	−0.002 (3)	0.009 (3)
C4	0.053 (3)	0.076 (4)	0.083 (4)	0.001 (3)	0.012 (3)	0.011 (3)
C5	0.068 (4)	0.101 (5)	0.088 (4)	0.004 (4)	−0.006 (3)	−0.003 (4)
C6	0.052 (3)	0.051 (3)	0.076 (4)	0.004 (3)	0.016 (3)	0.012 (3)
C7	0.058 (3)	0.065 (4)	0.064 (3)	0.012 (3)	0.011 (3)	0.016 (3)
C8	0.061 (3)	0.055 (3)	0.058 (3)	0.001 (3)	0.001 (3)	0.002 (3)
C9	0.075 (6)	0.044 (5)	0.118 (8)	0.006 (4)	0.013 (6)	0.011 (5)
C10	0.077 (4)	0.105 (6)	0.108 (5)	0.011 (4)	−0.007 (4)	0.002 (4)
N1	0.063 (3)	0.052 (3)	0.057 (3)	0.013 (2)	0.027 (2)	0.010 (2)
N2	0.063 (2)	0.050 (2)	0.048 (2)	0.009 (2)	0.015 (2)	0.0008 (19)
N3	0.074 (3)	0.037 (2)	0.068 (3)	0.019 (2)	0.017 (2)	0.008 (2)
N4	0.071 (3)	0.036 (2)	0.067 (3)	0.007 (2)	0.020 (2)	0.010 (2)
Cl1	0.0564 (7)	0.0618 (8)	0.0825 (9)	0.0211 (6)	0.0061 (7)	0.0030 (7)
Cl2	0.0655 (7)	0.0368 (6)	0.0575 (7)	0.0104 (5)	0.0234 (6)	0.0046 (5)
Cl3	0.0490 (6)	0.0764 (9)	0.0646 (8)	−0.0031 (6)	0.0088 (6)	−0.0196 (7)
Cl4	0.0670 (7)	0.0450 (7)	0.0610 (7)	0.0097 (6)	0.0157 (6)	0.0124 (6)

### Geometric parameters (Å, °)

**Table:**

Hg1—Cl1	2.4568 (14)	C7—C8	1.320 (7)
Hg1—Cl2	2.4811 (13)	C7—N4	1.353 (6)
Hg1—Cl4	2.4885 (14)	C7—H7	0.9300
Hg1—Cl3	2.4936 (15)	C8—N3	1.366 (6)
C1—N1	1.312 (6)	C8—H8	0.9300
C1—N2	1.325 (5)	C9—C10	1.402 (10)
C1—C4	1.490 (6)	C9—H9A	0.9700
C2—C3	1.332 (6)	C9—H9B	0.9700
C2—N2	1.366 (6)	C9'—C10	1.41 (3)
C2—H2	0.9300	C9'—H9C	0.9700
C3—N1	1.373 (6)	C9'—H9D	0.9700
C3—H3	0.9300	C10—H10A	0.9600
C4—C5	1.494 (7)	C10—H10B	0.9600
C4—H4A	0.9700	C10—H10C	0.9600
C4—H4B	0.9700	C10—H10D	0.9600
C5—H5A	0.9600	C10—H10E	0.9601
C5—H5B	0.9600	C10—H10F	0.9600
C5—H5C	0.9600	N1—H1N	0.8602
C6—N4	1.317 (6)	N2—H2N	0.8606
C6—N3	1.327 (6)	N3—H3N	0.8598
C6—C9	1.519 (10)	N4—H4N	0.8599
C6—C9'	1.55 (3)		
			
Cl1—Hg1—Cl2	115.95 (5)	C10—C9'—H9C	109.3
Cl1—Hg1—Cl4	105.47 (5)	C6—C9'—H9C	109.3
Cl2—Hg1—Cl4	112.40 (4)	C10—C9'—H9D	109.3
Cl1—Hg1—Cl3	106.12 (5)	C6—C9'—H9D	109.3
Cl2—Hg1—Cl3	105.11 (4)	H9C—C9'—H9D	108.0
Cl4—Hg1—Cl3	111.73 (5)	C9—C10—C9'	32.0 (9)
N1—C1—N2	106.7 (4)	C9—C10—H10A	109.5
N1—C1—C4	125.2 (4)	C9'—C10—H10A	117.4
N2—C1—C4	128.1 (5)	C9—C10—H10B	109.5
C3—C2—N2	106.8 (4)	C9'—C10—H10B	77.9
C3—C2—H2	126.6	H10A—C10—H10B	109.5
N2—C2—H2	126.6	C9—C10—H10C	109.5
C2—C3—N1	106.4 (4)	C9'—C10—H10C	126.8
C2—C3—H3	126.8	H10A—C10—H10C	109.5
N1—C3—H3	126.8	H10B—C10—H10C	109.5
C1—C4—C5	113.8 (5)	C9—C10—H10D	103.4
C1—C4—H4A	108.8	C9'—C10—H10D	109.5
C5—C4—H4A	108.8	H10A—C10—H10D	8.0
C1—C4—H4B	108.8	H10B—C10—H10D	107.7
C5—C4—H4B	108.8	H10C—C10—H10D	117.0
H4A—C4—H4B	107.7	C9—C10—H10E	137.6
C4—C5—H5A	109.5	C9'—C10—H10E	109.5
C4—C5—H5B	109.5	H10A—C10—H10E	106.2
H5A—C5—H5B	109.5	H10B—C10—H10E	35.0
C4—C5—H5C	109.5	H10C—C10—H10E	78.5
H5A—C5—H5C	109.5	H10D—C10—H10E	109.5
H5B—C5—H5C	109.5	C9—C10—H10F	83.0
N4—C6—N3	105.3 (4)	C9'—C10—H10F	109.5
N4—C6—C9	123.8 (5)	H10A—C10—H10F	104.5
N3—C6—C9	130.2 (6)	H10B—C10—H10F	136.5
N4—C6—C9'	131.4 (12)	H10C—C10—H10F	31.4
N3—C6—C9'	117.9 (11)	H10D—C10—H10F	109.5
C9—C6—C9'	29.3 (9)	H10E—C10—H10F	109.5
C8—C7—N4	107.3 (5)	C1—N1—C3	110.2 (4)
C8—C7—H7	126.3	C1—N1—H1N	124.9
N4—C7—H7	126.3	C3—N1—H1N	124.9
C7—C8—N3	106.1 (5)	C1—N2—C2	109.9 (4)
C7—C8—H8	126.9	C1—N2—H2N	125.1
N3—C8—H8	126.9	C2—N2—H2N	125.0
C10—C9—C6	114.1 (6)	C6—N3—C8	110.5 (4)
C10—C9—H9A	108.7	C6—N3—H3N	125.1
C6—C9—H9A	108.7	C8—N3—H3N	124.4
C10—C9—H9B	108.7	C6—N4—C7	110.8 (4)
C6—C9—H9B	108.7	C6—N4—H4N	124.7
H9A—C9—H9B	107.6	C7—N4—H4N	124.5
C10—C9'—C6	111.5 (18)		

### Hydrogen-bond geometry (Å, °)

**Table:**

*D*—H···*A*	*D*—H	H···*A*	*D*···*A*	*D*—H···*A*
N1—H1N···Cl3^i^	0.86	2.42	3.227 (4)	157
N2—H2N···Cl4^ii^	0.86	2.37	3.188 (4)	158
N3—H3N···Cl2	0.86	2.37	3.220 (4)	171
N4—H4N···Cl2^iii^	0.86	2.47	3.285 (4)	159

Symmetry codes: (i) *x*, *y*+1, *z*+1; (ii) −*x*+2, −*y*+1, −*z*+1; (iii) *x*, *y*+1, *z*.
